# Integrating Cancer Vaccines in the Standard-of-Care of Ovarian Cancer: Translating Preclinical Models to Human

**DOI:** 10.3390/cancers13184553

**Published:** 2021-09-10

**Authors:** Cheryl Lai-Lai Chiang, Raphaël Rovelli, Apostolos Sarivalasis, Lana E. Kandalaft

**Affiliations:** 1 Department of Oncology, Centre Hospitalier Universitaire Vaudois (CHUV), University of Lausanne, CH-1011 Lausanne, Switzerland; raphael.rovelli@unil.ch (R.R.); Apostolos.Sarivalasis@chuv.ch (A.S.); 2 Ludwig Institute for Cancer Research, University of Lausanne, CH-1066 Lausanne, Switzerland; 3 Center of Experimental Therapeutics, Department of Oncology, Centre Hospitalier Universitaire Vaudois (CHUV), CH-1011 Lausanne, Switzerland

**Keywords:** ovarian cancer, cancer vaccines, combinatorial immunotherapy strategies, tumor microenvironment, antitumor responses

## Abstract

**Simple Summary:**

The overall survival of ovarian cancer (OC) remains poor for most patients. Despite incorporation of novel therapeutic agents such as bevacizumab and PARP inhibitors to OC standard-of-care, efficacy is only observed in a subset of patients. Cancer vaccination has demonstrated effectiveness in OC patients and could be considered for potential incorporation into OC standard-of-care. This review provides an overview of the different types of cancer vaccination strategies and discusses the use of murine OC tumor models to evaluate combinatorial regimens comprising cancer vaccines and OC standard-of-care.

**Abstract:**

As the majority of ovarian cancer (OC) patients are diagnosed with metastatic disease, less than 40% will survive past 5 years after diagnosis. OC is characterized by a succession of remissions and recurrences. The most promising time point for immunotherapeutic interventions in OC is following debulking surgery. Accumulating evidence shows that T cells are important in OC; thus, cancer vaccines capable of eliciting antitumor T cells will be effective in OC treatment. In this review, we discuss different cancer vaccines and propose strategies for their incorporation into the OC standard-of-care regimens. Using the murine ID8 ovarian tumor model, we provide evidence that a cancer vaccine can be effectively combined with OC standard-of-care to achieve greater overall efficacy. We demonstrate several important similarities between the ID8 model and OC patients, in terms of response to immunotherapies, and the ID8 model can be an important tool for evaluating combinatorial regimens and clinical trial designs in OC. Other emerging models, including patient-derived xenograft and genetically engineered mouse models, are continuing to improve and can be useful for evaluating cancer vaccination therapies in the near future. Here, we provide a comprehensive review of the completed and current clinical trials evaluating cancer vaccines in OC.

## 1. Current Ovarian Cancer Standard-of-Care and Limitations

Ovarian cancer (OC) remains the deadliest gynecological malignancy and the eighth most common cancer-related death in women worldwide [1]. In the United States, approximately 13,770 women will die from OC in 2021 [2]. OC overall survival is poor due to frequent late-stage diagnosis at presentation—a consequence of mild or no specific symptoms of the disease in early stage. The first-line, standard-of-care treatments of OC are debulking surgery and platinum doublet chemotherapy. Debulking surgery is performed to ensure maximal resection of all visible tumors and for tumor staging, and optimal debulking is often defined as <1 cm residual tumor per nodule [3]. It has been shown that OC patients experienced better prognosis in the absence of macroscopic residual disease [4,5,6]. Patients with advanced disseminated disease or poor clinical status can be treated with neoadjuvant chemotherapy prior to debulking surgery [7]. For the past 30 years, platinum and paclitaxel-based chemotherapy have been considered the standard first-line drugs for treating OC. In two large-scale trials, patients treated with cisplatin and paclitaxel experienced an 11% survival advantage over patients treated with cisplatin and cyclophosphamide, and 40% of the patients in the former remained alive after 5 years [8,9,10]. As an alternative to intravenous (iv) chemotherapy, intraperitoneal (ip) chemotherapy can be given to achieve a more localized anti-tumor activity [10,11].

### 1.1. Maintenance Therapies

Following first-line surgery and chemotherapy, OC patients may receive maintenance therapies. Bevacizumab, a humanized monoclonal antibody that binds to circulating vascular endothelial growth factor (VEGF)-A isoform, was approved by the US Food and Drug Administration (FDA) and European Medicines Agency (EMA) in 2011 and 2018, respectively. It is used in combination with carboplatin and paclitaxel as first-line and maintenance therapy in advanced stage epithelial ovarian, fallopian tube and primary peritoneal cancers [12,13]. VEGF-A plays an important role in inducing tumor angiogenesis and supporting tumor growth in OC and many types of cancers. By targeting VEGF-A, bevacizumab inhibits its interaction with VEGF receptor, thereby preventing tumor angiogenesis [14]. OC patients who showed increased levels of VEGF in their sera also experienced poorer survivals [15,16,17]. Inhibiting VEGF also helps to normalize tumor vasculature for enhanced chemotherapeutic drug delivery and tumor toxicity as well as to reduce ascites fluid formation through reducing tumor vasculature permeability in OC [18,19].

Recently, poly-adenosine diphosphate (ADP)-ribose-polymerase (PARP) inhibitors are used as a maintenance therapy in OC. High-grade serous ovarian carcinoma (HGSOC) is the commonest and most lethal OC sub-type [20]. About 20% of HGSOCs harbor germline mutation in breast cancer genes *BRCA1* or *BRCA2* [21,22,23]. These genes are of paramount importance for repairing deoxyribonucleic acid (DNA) double-strand breaks (DSB) via homologous recombination (HR) mechanisms [24]. Overall, up to 50% of HGSOCs has shown HR deficiencies (HRD) due to germline *BRCA* gene mutations, somatic *BRCA1/2* gene mutations, epigenetic loss of *BRCA1* gene, *BRCA* gene promoter methylation and other HR deficiency mechanisms [25,26]. PARP inhibitors are a particularly effective maintenance treatment in OC, especially in HGSOC displaying HRD phenotype. These small molecules mimic nicotinamide binding at the NAD^+^, leading to further disruption in DNA repair and genomic arrest in cancer cells [27,28,29]. FDA and EMA have approved three PARP inhibitors (olaparib, niraparib and rucaparib) as maintenance therapies in platinum-sensitive recurrent OC [27,28,30,31,32,33,34]. Based on the results of two recent phase III trials PAOLA-1 [35] and PRIMA [36], FDA and EMA approved the combination use of olaparib and bevacizumab and niraparib monotherapy as first-line maintenance therapies in recurrent OC. Notably, niraparib is approved for use in advanced OC regardless of BRCA status [36]. These approvals extended the use of olaparib and generally PARP inhibitors as a maintenance monotherapy beyond BRCA mutated HGSOC [37].

### 1.2. Disease Recurrence and Side Effects from Standard-of-Care

Although most OC patients respond well to first-line treatments, 80% will eventually recur and 60% will die within 5 years of diagnosis [38]. Platinum-sensitive OC patients (defined as those who respond to platinum-based chemotherapy) will typically recur >6–12 months after the last chemotherapy dose, while platinum-resistant OC patients will recur in <6 months following the last dose [20]. Such platinum-resistant patients will be treated with chemotherapeutic agents, including pegylated liposomal doxorubicin, topotecan, gemcitabine and paclitaxel alone or in combination with bevacizumab [39]. In the Aurelia phase III trial, combining chemotherapy and bevacizumab led to a modest 3.3-month increase in the median progression-free survival (PFS) when compared to chemotherapy alone (6.7 months versus 3.4 months) [39]. Other limitations in the current OC standard-of-care chemotherapeutic drugs include serious side effects such as fatigue, nausea, vomiting, leukopenia, thrombocytopenia, anemia and hypertension that can lower patient compliance. Moreover, acquired resistance to chemotherapeutic drugs is a significant issue [40,41,42]. Bevacizumab can induce rare serious side effects, including hypertension, wound healing impairment, thrombosis, severe bleeding, colon perforations, and fistulas causing severe infections [12,13]. Similarly, PARP inhibitors can cause hematological toxicities, constitutional symptoms including fatigue, digestive disturbances and rarely pneumonitis and blood malignancies such as myelodysplastic syndrome and acute myeloid leukemia [35]. The reversal of *BRCA1/2* gene mutations in OC has been associated with resistance to PARP inhibition and platinum chemotherapy [43,44]. Thus, effective new OC treatment regimens are urgently needed.

## 2. Cancer Vaccine Strategies

Cancer immunotherapy, the fifth pillar of core cancer treatment after surgery, chemotherapy, targeted therapies and radiotherapy, can potentially be integrated into OC standard-of-care. OC is a good candidate for immunotherapy as several studies have consistently demonstrated a positive correlation between an increased presence of CD3^+^ tumor-infiltrating lymphocytes (TILs) and increased overall survivals [45,46,47,48]. These observations strongly supported the notion that OC is immunogenic and T cells play an important role in suppressing OC. Cancer vaccination emerges as an attractive approach to activate endogenous T cells to destroy tumors and induce long-term immunological memory against OC.

OC is a highly heterogeneous disease and is broadly classified into five major subtypes: endometroid carcinoma (EM), clear cell carcinoma (CCC), HGSOC, low-grade serous carcinoma (LGSOC) or mucinous carcinoma (MOC), based on the revised World Health Organization 2014 criteria [49]. They differ in pathogenesis, origin, molecular alterations and prognosis. For example, the EM, CCC, LGSOC and MOC subtypes are mainly characterized by the activation of ERRB2/KRAS/BRAF/MEK, Wnt and PI3K/AKT signaling pathways. These subtypes also exhibit inactivation in the PTEN pathway and ARID1A-related chromatin remodeling [50]. Conversely, HGSOC shows ubiquitous *TP53* gene mutation and activation in the Notch 3, FOXM1 and cyclin E1 signaling pathways [50]. Alterations in p53 and cycline E1 pathways also indirectly contribute to genome instability in HGSOC, which is an essential molecular feature of this subtype. Moreover, approximately 20% of HGSOC has *BRCA1* or *BRCA2* gene mutations that render it sensitive to PARP inhibition [21,22,23]. It is therefore reasonable to postulate that each OC subtype can possess a distinct set of tumor antigens arising from different molecular and genetic alterations. Individual patients with the same OC subtypes may also express unique tumor neoantigens. This has important implications in cancer vaccine development as the use of autologous personalized cancer vaccines may lead to more effective tumor targeting in individual patients. Here, we discuss the different types of cancer vaccination approaches that are potentially applicable to OC. We also perform a search on the website www.clinicaltrials.gov (accessed on 2 September 2021) for completed and current OC trials that evaluate cancer vaccine strategies and summarize them in Table 1 and Table 2, respectively.

### 2.1. Tumor-Associated Antigen and Tumor Neoantigen Peptide Vaccines

In the past decades, major advancements in molecular, proteomics and serological techniques have assisted in identifying numerous shared tumor-associated antigens (TAAs) in different tumor types. Shared TAAs are essentially self-antigens and can be broadly classified into four major categories [99,100]: (1) normal proteins that are overexpressed in tumors (e.g., HER-2/neu (human epidermal growth factor receptor 2), MUC1 (mucin 1) and WT1 (Wilms tumor 1) in OC); (2) lineage-specific differentiation TAAs (e.g., MART-1(melanoma-associated antigen recognized by T cells-1) and gp100 (glycoprotein 100) in melanoma); (3) aberrantly expressed in tumors but with restricted expression in normal testis (e.g., cancer-testis antigens NY-ESO-1 (New York esophageal squamous cell carcinoma-1), synovial sarcoma X chromosome; (4) oncofetal antigens that are expressed on embryonic or fetal tissues (e.g., CEA (carcinoembryonic antigen) in colorectal carcinoma). Synthetic TAA peptides can be easily produced in large quantities for clinical trial use. However, most of the identified TAAs are derived from human leukocyte antigen (HLA)-A2-restricted patients, therefore precluding the use in patients with other HLA haplotypes. Another major concern is that targeting one TAA may be insufficient to eradicate the tumor and can even lead to tumor cell escape. Nevertheless, numerous OC clinical trials using TAAs synthetic peptides have been initiated and produced clinical benefits in certain patients (Table 1 and Table 2).

In contrast to shared TAAs, tumor neoantigens are expressed exclusively on tumor cells (reviewed in [101]). They are private ‘non-self’ mutated tumor antigens derived either directly from transformation processes (driver mutations) or from genomic instability caused by increase genome alternations during tumor cell division (passenger mutations) [102]. As tumor neoantigens are considered ‘non-self’, they are not subjected to thymic selection and central tolerance similar to TAAs [103] and may be able to elicit high-avidity neoantigen-reactive T cells. Wölfel and colleagues demonstrated that cytotoxic T lymphocytes (CTLs) can recognize a non-synonymous mutation in cyclin-dependent kinase 4 in melanoma [104]. In OC, transient neoantigen-reactive CD8^+^ T cells have been detected [105]. Although OC is considered a low mutation burden cancer [106], we could identify immunogenic tumor neoantigens in OC patients following dendritic cell (DC) vaccinations [96]. We also detected CD4^+^ and CD8^+^ T cell responses directed against a pool of neoantigen peptides in an OC patient [97]. Using the murine ID8 ovarian model, we and Martin et al. identified numerous tumor neoantigens [97,107]. We further demonstrated that an increasing number of specific tumor neoantigen-reactive T cells elicited by DC vaccination is positively correlated with a reducing tumor growth in this model [97]. Hence, these demonstrated the feasibility of targeting tumor neoantigens in OC.

The use of next-generation sequencing (NGS) and sophisticated neoepitope prediction algorithms have helped to advance the discovery of tumor neoantigens [108,109]. Following whole-exome sequencing of a patient’s tumor biopsy, mutation sequences are analyzed with a neoepitope prediction algorithm for in silico binding to human leukocyte antigen (HLA) Class I molecules. Candidate neoantigens are then ranked and may be tested with in vitro binding assays to further refine the selection. Single nucleotide variants (SNVs) that result from a single nucleotide substitution are commonly used for identifying tumor neoantigens. Other mutations, including frameshifts, insertion-deletions (indels) and chromosomal translocations can produce tumor neoantigens with higher affinities to HLA molecules caused by larger sequence divergences and should be investigated [110,111,112]. Targeting driver mutations that provide intrinsic tumor growth advantages may be important, as well as clonal or truncal tumor neoantigens expressed by every tumor cell [113]. Conversely, targeting subclonal or branch mutations present in a subset of tumor cells is insufficient in eradicating tumors [114]. Currently, there is no consensus on a standard neoepitope prediction algorithm for use in clinics [115,116]. It is reported that less than 3% of the identified tumor neoantigens successfully elicited T cell responses in patients [117], emphasizing the urgent need for more accurate predication algorithms. New methods are being developed, including a proteogenomic strategy that uses a high-throughput mass spectrometry platform to identify tumor-specific antigens from non-coding sequences, which yielded interesting sequences derived from epigenetic changes in atypical translation events and sequences from mutations [118].

Tumor neoantigens can be synthesized as short or long peptides for vaccination. Peptides of nine amino-acid residues bind directly to HLA Class I molecules to activate CD8^+^ T cells. Longer peptides of up to 30 amino acid residues are internalized by antigen-presenting cells (APCs) such as DCs for further processing and presenting on HLA molecules. These longer peptides may activate CD4^+^ helper and CD8^+^ effector T cells, inducing memory responses [119]. A search on www.clinicaltrials.gov (accessed on 2 September 2021) revealed only one clinical trial utilized tumor neoantigens for vaccination in OC (Table 2). In this trial (NCT04024878), thirty OC patients will be recruited and vaccinated with ~20 tumor neoantigen peptides (five vaccinations and two boosters) identified from their tumors. Poly-ICLC (Hiltonol), a Toll-like receptor (TLR) 3 agonist to simulate interferon (IFN) secretions from DCs [120], will be used as an adjuvant and co-administered intradermally with the neoantigen peptides. The patients will also receive nivolumab, an anti-programmed death (PD)-1 antibody, intravenously over 2 weeks. Such a neoantigen-based vaccination strategy has demonstrated effectiveness in melanoma and glioblastoma. In a melanoma trial, 10 patients were vaccinated with 13–20 neoantigen peptides with Poly-ICLC (five vaccines and two boosters) [121]. The treatment was well tolerated, and adverse events were limited to mild flu-like symptoms and fatigue. Polyfunctional vaccine-primed CD4^+^ and CD8^+^ T cells were elicited, and four patients showed no disease recurrence 25 months post-vaccination [121]. In a phase I/Ib trial of 10 newly diagnosed glioblastoma patients, polyfunctional neoantigen-specific CD4^+^ and CD8^+^ T cells with memory phenotype were detected following vaccination, with up to 20 neoantigen peptides in combination with Poly-ICLC [122]. Thus, the trial results from OC shall be highly anticipated.

### 2.2. DNA and RNA Vaccines

DNA and RNA (ribonucleic acid) vaccines have shown good safety and immunogenicity profiles in cancers and other diseases [123,124]. Only a small number of a patient’s tumor cells is needed to generate the vaccines. The extracted tumor DNA and RNA can be easily amplified by polymerase chain reaction to scale up vaccine production. This is especially useful when patients’ materials are limited. DNA vaccines are developed from bacterial plasmid modified to express specific genes of interest. Genes encoding for cytokines (e.g., IL-2, granulocyte-macrophage colony stimulating factor (GM-CSF)) and/or costimulatory molecules (e.g., CD28, 4-1BB) can be added to the DNA vaccine to increase its effectiveness. DNA vaccines can be given via different routes including intramuscularly, intradermally or subcutaneously for the plasmids to enter the cell nuclei of the transfected cells to initiate expression of the desired genes. DNA vaccines can elicit CD8^+^ T cells [125], humoral [126] and memory responses [127,128]. RNA vaccines can be administered via the same routes as DNA vaccines, as wells as into the lymph nodes, organs or via a nasal spray, depending on the formulation. RNA vaccine can also be electroporated or pulsed onto DCs for antigen expression, processing, and presenting [129]. Unlike DNA that requires cell nuclei machinery for its expression, RNA is translated into functional proteins in the cell cytoplasm hence avoiding the risk of it integrating into host cell genome.

Few clinical trials have evaluated DNA plasmid vaccines encoding for tumor antigens in OC (Table 1). A study described the use of a multi-neoantigen DNA plasmid vaccine in different tumor models, including the murine ID8 ovarian model [130]. The neoepitopes were identified by comparing the sequencing of cell lines cultured in vitro to the same cell lines that were implanted into mice. A total of 27 nonsynonymous expressed mutations were identified in the ID8 model, and 24 neoepitopes were selected for in vivo vaccination following in silico evaluation with NetMHCons v1.1. This DNA vaccine elicited a predominant CD8^+^ T cell response and significantly increased the overall survival of ID8 tumor-bearing mice in the prophylactic setting [130]. There is no OC trial that has evaluated or currently evaluating personalized neoepitope DNA/RNA vaccines on www.clinicaltrials.gov (accessed on 2 September 2021). Ugur and colleagues conducted a first-in-human study to evaluate a personalized neoantigen RNA vaccine in melanoma [131]. Thirteen stage III/IV-melanoma patients were evaluated, and up to 10 mutations were selected for each patient to create personalized RNA vaccines encoding for 27mer neopeptides [131]. Each patient received up to 20 vaccine doses without serious adverse effects. T cells specific to at least three mutations were elicited in each patient, and pre-existing responses against certain neoepitopes were augmented. One-quarter of the neoepitopes elicited both CD4^+^ and CD8^+^ T cells recognizing different regions of the mutated 27mer neopeptides [131]. A sustained PFS was observed in the patients, and two out of five patients with metastatic disease achieved objective responses. One patient had a complete response when also given anti-PD-1 therapy. In another study, RNA vaccine was used to elicit neoantigen-specific T cells in four patients with metastatic gastrointestinal cancer [132]. Using high-throughput screenings with long peptides and tandem minigenes covering all mutated epitopes, neoepitopes recognized by autologous TILs were identified, and up to 15 were used in the RNA vaccine encoding for 25mer neopeptides [132]. Mutations in the *TP53*, *KRAS*, or *PIK3CA* driver genes were also included in the vaccine. Up to eight RNA vaccine doses were given with no adverse effect. Although no objective clinical responses were observed in all the patients, vaccine-induced neoantigen-reactive T cells were detected. These initial studies demonstrated that such a personalized RNA vaccine strategy is feasible and warrants evaluation in OC. RNA vaccine can be further enhanced with immune checkpoint blockade (ICB) therapy [131].

### 2.3. Viral Vector Vaccines

Viral vector vaccines have gained interest in cancer immunotherapy as many viruses are naturally immunogenic and highly capable of infecting mammalian cells. Viral vector vaccines can be created by genetically modifying the genome of viral particles to express specific genes of interest. The advantages and disadvantages of different viral vectors have been reviewed [133] and are not discussed here. Viral vectors from the poxviridae family, including vaccinia virus, fowl pox and canarypox, are extensively investigated in cancer immunotherapy. These doubled-stranded DNA viruses are able to pack large foreign gene inserts and show the ability to infect a broad host range [133]. An advantage is that viral vector vaccines can potentially be manufactured as an ‘off-the-shelf’ formulation, as they are highly stable for a long period. A major challenge is to overcome the development of host-induced neutralizing antibodies to the viral vector itself, as this can significantly impede its use for repeat vaccination in the patients.

A number of OC clinical trials have evaluated viral vector vaccines targeting known OC TAAs (see also Table 1). Recombinant vaccinia (PANVAC-V) and fowl pox (PANVAC-F) vector vaccines were engineered to express carcinoembryonic antigen, MUC1, and TRIad of COstimulatory Molecules (B7-1/ICAM-1/LFA-3, designated TRICOM [134]) and used as primary and booster vaccines, respectively [135]. A median PFS of 18 months and median OS of 19 months were observed in the OC patients [135]. In another study, recombinant vaccinia and fowl pox encoding NY-ESO-1 (i.e., rV-NY-ESO-1 and rF-NY-ESO-1) were generated to treat patients with NY-ESO-1-positive tumors [136]. Patients were vaccinated with either of the viral vector vaccines or both. An OC patient was disease-free for 8 months after vaccination [136]. Another study also utilized rV-NY-ESO-1 and rF-NY-ESO-1 to treat advanced OC and melanoma patients [92]. Nine out of 22 OC patients demonstrated humoral responses, and 15 patients showed CD4^+^ T cell responses. Finally, a modified Vaccinia Ankara vaccine encoding wild-type p53 antigen was used to vaccinate platinum-resistant OC patients [82]. Five of the 11 patients showed increased p53-specific T cells, and the median PFS of responders and non-responders were 7 and 2.3 months, respectively [82].

### 2.4. DC-Based and Whole Tumor Cell-Based Vaccines

DCs are specialized cells in the immune system and act as a bridge between innate and adaptive immunities [137]. These professional APCs are among the first-responders to eliminate pathogens and to take part in tissue repair and homeostasis [138]. As DCs are critical for modulating immune responses, DC-based immunotherapy has been actively investigated in many cancers, including OC. Different subsets of DCs exist and are distinguished by their phenotypic markers, tissue locations and the immune responses they elicit [139]. They are highly apt in modulating local tissue immune responses [139]. Monocyte-derived DCs are the commonest DC subset investigated in clinics and are easily generated in vitro by culturing peripheral blood monocytes with recombinant IL-4 and GM-CSF. Here, we discuss the use of this DC subset in OC immunotherapy.

Whole tumor cell lysate (WTL) is an attractive antigen source for DCs as autologous tumor cells can easily be recovered during OC debulking surgery. Autologous WTL can encompass all antigens present in tumors, including shared TAAs and private mutated neoantigens. Previously, we used hypochlorous acid (HOCl) to induce oxidation and rapid necrosis of tumor cells for WTL preparation. HOCl is a potent microbicidal agent and a strong oxidant that can increase the immunogenicity of protein antigens [140,141,142]. We demonstrated in an ID8-ovalbumin (ID8-OVA)-expressing model that DCs pulsed with HOCl-oxidized ID8-OVA-WTL significantly prolonged the survival of tumor-bearing mice [143]. We further demonstrated that heavily pretreated recurrent OC patients vaccinated with autologous DC-oxidized autologous WTL vaccine (called OCDC) developed polyclonal T cell responses against known OC TAAs [143], as well as de novo T cell responses against previously unrecognized private tumor neoantigens [96]. OCDC vaccination led to priming of significantly higher avidity (~100-fold increase) T cells against previously recognized neoepitopes, and the elicited T cell responses were associated with prolonged PFS in the patients (*p* = 0.05) [96]. These results supported the use of personalized DC-WTL vaccine to elicit neoantigen-specific T cells in OC. In a proposed randomized phase I/II study in advanced OC (Swissmedic reference number 2019TpP1004), we will compare the effectiveness of OCDC to DCs pulsed with patient-derived neoantigen peptides (up to 10 neoepitopes) given intranodally. Patients will also receive low-dose iv cyclophosphamide [144]. The immunogenicity and safety of the vaccines will be evaluated, as well as the PFS and OS of the OC patients for up to 36 months [144]. Alternatively, DC-fusion vaccine can be created by fusing autologous DCs with autologous tumor cells therefore bypassing the need to pulse DCs ex vivo [NCT00799110].

The feasibility of using modified autologous tumor cells as cancer vaccines has been investigated. In a phase I/II trial, patients with solid tumors including OC patients were vaccinated intradermally with autologous tumor cells modified to express GM-CSF and a bifunctional short hairpin RNAi (bi-shRNAi) targeting furin convertase to downregulate endogenous transforming growth factors (TGF)-β1 and -β2 (Vigil/FANG vaccine) [145]. Adverse events were limited to grade 1 and 2, and vaccine-induced T cell responses were detected in 9 out of the 18 patients and correlated with prolonged survival [145]. Three out of the five OC patients in the study showed stable diseases [145]. A phase II trial was conducted to evaluate Vigil vaccine as a maintenance therapy in stage III/IV OC [93]. Of the 42 patients enrolled, 31 received the Vigil vaccines and 11 received standard-of-care as controls. Increased vaccine-induced T cells were correlated with prolonged PFS (median 604 days versus median 377 days in the control; *p* = 0.033) [93]. In a randomized, placebo-controlled, phase IIb trial, 91 patients received either Vigil vaccine (Gemogenovatucel-T) (*n* = 47) or placebo (*n* = 44) intradermally for a minimum of 4 and up to 12 doses [146]. PFS in patients receiving Vigil vaccine was 11.5 months as compared to 8.4 months in the placebo group (*p* = 0.078) [146]. The authors concluded that front-line use of Vigil was well tolerated but did not help to prolong PFS in this trial, and they proposed to evaluate OC patients based on their BRCA mutation status. Other clinical trials using WTL/tumor cells as cancer vaccines in OC are summarized in Table 1 and Table 2.

## 3. Integrating Cancer Vaccines into OC Standard-of-Care Regimen

**Figure 1:**
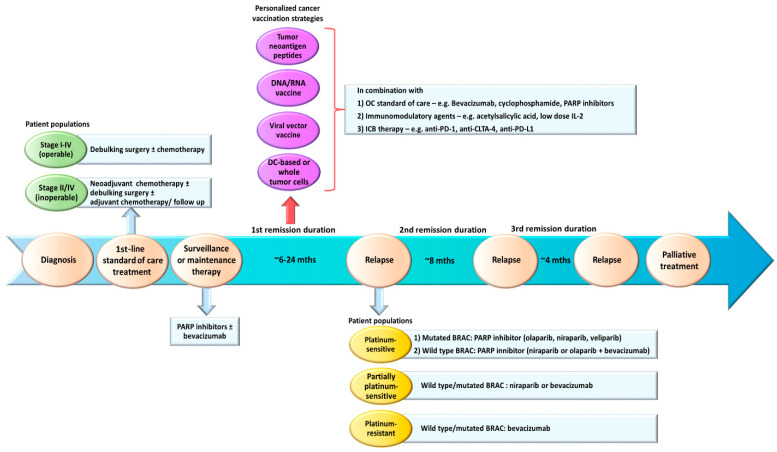
An overview of the general standard-of-care treatments in OC and proposed personalized cancer vaccination strategies in the first remission after first-line treatments. After diagnosis, OC patients with operable diseases will undergo debulking surgery and chemotherapy. Patients with early stage I disease usually have a good outcome after first-line treatments. Patients who are inoperable (e.g., old age, poor health or extensive metastasis) will receive neoadjuvant chemotherapy, and if downstaged sufficiently, can be assessed for a debulking surgery with subsequent adjuvant chemotherapy. Then, advanced stage III and IV patients will be offered maintenance therapy consisting of PARP inhibitors ± bevacizumab. Most patients will relapse within 24 months after the first-line treatment (first remission). Based on their responsiveness to platinum chemotherapy and BRCA (wild type/mutated) status, they will be stratified to received different types of PARP inhibitors or bevacizumab. We proposed to introduce personalized cancer vaccination strategies (e.g., tumor neoantigen peptides, DNA, RNA or viral vector vaccines, DC-based or modified whole tumor cell vaccines) during the first remission when the patients are presented with minimal disease (i.e., complete respond (CR) or no evidence of disease (NED)) after first-line treatments. OC standard of care, such as bevacizumab, cyclophosphamide and PARP inhibitors can potentially be incorporated in the strategy. In addition, ICB therapy (e.g., anti-PD-1, anti-CTLA-4 and anti-PD-L1) and other immunomodulating agents (e.g., acetylsalicylic acid and low-dose IL-2) can be considered to help augment vaccine-primed antitumor T cell responses and modulate the OC TME.

Currently, most patients will recur within 3 years after first-line treatments and will require further rounds of chemotherapy and a maintenance therapy with bevacizumab and/or iPARP. Hence, OC is characterized by successive periods of oncological remission and recurrence with the time to progression drastically reduced with each recurrence [20]. We consider the period in the aftermath of the debulking surgery and the end of the primary chemotherapy as the best time frame to administer the cancer vaccines (Figure 1). This period may last from 6 to >24 months depending on the patient’s sensitivity to platinum chemotherapy and BRCA status [20]. During this period, the patients will have minimal residual diseases, making them ideal candidates for cancer vaccinations. The OC tumor microenvironment (TME) may also become less immunosuppressive due to the destruction of tumor cells and tumor vasculatures by chemotherapy. Studies have shown that certain chemotherapy can induce immunogenic tumor cell death, leading to the activation of tumor-specific T cells. Doxorubicin, an anthracycline chemotherapy used in OC, may induce immunogenic apoptotic tumor cell death and caspase activation in murine CT26 colon carcinoma and B16.F10 melanoma models [147]. DCs phagocytosed doxorubicin-treated tumor cells and successfully elicited CD8^+^ T cells to suppress tumor growth [147]. In OC, patients who underwent debulking and platinum-taxane chemotherapy developed memory T cells that recognized OC antigens and experienced prolonged survival [148]. Moreover, a study demonstrated a positive correlation between the potency of CD8^+^ T-cell responses following chemotherapy and favorable clinical outcome [149]. Platinum and taxane chemotherapy used in OC have shown to exert immunomodulatory effects [150,151]. These favorable features can help to strengthen the ability of cancer vaccines in activating potent antitumor T cell responses and developing immunological memory for durable tumor control.

The human omentum plays a central role in peritoneal homeostasis, including tissue repair, angiogenesis, nutrients transportation, and lipid storage as well as fighting infections (reviewed in [152]). It is comprised of adipose tissues with intertwined networks of blood vessels, stromal cells and connective matrix components. Importantly, the omentum contains lymphoid aggregates called milky spots (MS) that help to elicit peritoneal immunity against invading pathogens and promoting inflammation or tolerance depending on the antigenic stimuli ([152]). The MS are formed particularly around glomerulus-like knots of blood vessels in the omentum, and CD4^+^ and CD8^+^ T cells are present alongside B cells, CD1d-restricted natural killer (NK)T cells, innate lymphoid cells (ILCs; particularly ILC2 population), and CD11c^+^ and CD11b^+^ myeloid cells ([152]). The MS serves as an important filer for the peritoneal fluid by capturing antigens or pathogens, initiating suitable immune responses. It will be advantageous to utilize cancer vaccines to activate adaptive immunity in the MS as well. Conversely, the omentum and MS have been shown to assist in OC tumor metastasis and progression (reviewed in [152,153]). Hence, combinatorial therapeutic approaches that not only activate antitumor immunity but also overcome such pro-tumor mechanisms are essential.

In a pilot study, we demonstrated that heavily pretreated recurrent OC patients can be successfully vaccinated with a personalized cancer vaccine (OCDC) [143]. These advanced stage patients received two to seven rounds of prior chemotherapy, and two out of the five patients achieved stable diseases following vaccination [143]. The patients were given five doses of OCDC (~5–10 × 10^6^ DCs/dose) intranodally every two weeks. OCDC vaccine was well tolerated and no severe adverse events were observed. Four weeks after the 5th vaccine, OCDC elicited T cells that recognized different TAAs including HER-2/neu and MUC1 expressed on the patients’ tumors. Two patients who entered the study with no evidence of disease experienced a longer second PFS after OCDC vaccination. Three other patients who entered the study with radiographically measurable disease progressed after OCDC vaccination; however, one of them experienced a regression or stabilization in 6 out of the 13 tumor metastatic deposits in a second follow up [143]. These results suggest that the immune system of heavily pretreated recurrent OC patients is not impaired by prior chemotherapy, and patients with measurable diseases may benefit from cancer vaccination. Similarly, the Vigil/FANG vaccine (modified autologous whole tumor cells) can induce stable diseases in three out of the five heavily pretreated OC patients [145]. Each patient received a vaccine dose (1 million cells/injection) once a month for up to 12 doses [145]. The Vigil/FANG vaccine was also evaluated as a maintenance therapy in advanced stage III/IV OC patients who were optimally or suboptimally debulked and had received several rounds of first-line chemotherapy [93]. The vaccine was able to elicit antitumor T cells and significantly prolong PFS in these patients (19.8 months as compared to 12.4 months in control patients) [93]. A 4-year PFS rate was achieved in 27.6% of the patients who received Vigil/FANG vaccine as compared to 9.1% of the control patients [154]. Hence, these studies showed that personalized cancer vaccines are safe and OC patients can respond to such cancer vaccinations regardless of their prior treatments.

To determine if cancer vaccination can be given in combination with OC standard-of-care, we evaluated OCDC with bevacizumab and cyclophosphamide in recurrent OC patients [96]. The patients were randomized into three different treatment cohorts to receive OCDC only, OCDC plus bevacizumab or OCDC, bevacizumab (15 mg/kg) every three weeks and cyclophosphamide 200mg/m^2^, iv weekly. Each patient received five doses of OCDC intranodally every two or three weeks. Cyclophosphamide was given one day prior to OCDC vaccination to deplete Treg cells, and bevacizumab on the day of OCDC vaccination to target VEGF [96]. Patients who received OCDC-bevacizumab-cyclophosphamide regimen showed a higher fold-expansion of OCDC-induced T cells as well as a transient increase in proinflammatory IFN-γ and decrease in immunosuppressive TGF-β in the sera when compared to patients who received OCDC only or OCDC plus bevacizumab. Moreover, 80% of the patients treated with OCDC-bevacizumab-cyclophosphamide regimen remained alive at 25 months post-treatment compared to 50% of patients treated with bevacizumab plus cyclophosphamide without OCDC [96]. The cancer vaccine can be used in combination with OC standard-of-care to achieve a greater efficacy in recurrent OC patients.

### Combining Cancer Vaccines with Immunomodulatory Agents

We expanded the treatment cohorts and provided evidence that the use of acetylsalicylic acid and low-dose IL-2 in the OCDC-bevacizumab-cyclophosphamide regimen can further enhance OCDC-primed antitumor T cells [97]. The aim of this study was to incorporate FDA-approved immunomodulatory agents into a regimen consisting of a personalized cancer vaccine (OCDC) and OC standard-of-care (bevacizumab and cyclophosphamide). Acetylsalicylic acid (325 mg of enteric-coated aspirin) was given from the first day of OCDC vaccination for up to 84 days, while low-dose IL-2 (2MIU/dose) was given for 5 consecutive days from the day of OCDC vaccination. Recurrent OC patients treated with this combinatorial strategy showed increased antitumor polyclonal T cell responses characterized by higher granzyme B, perforin, TNF-α and IFN-γ expressions as well as a higher 3 year overall survival rate (80%) when compared to patients who did not receive additional acetylsalicylic acid and low-dose IL-2 (40%) [97]. Analysis in the murine ovarian ID8 tumor model revealed that this combinatorial strategy was able to modulate the OC TME to improve the activation of antitumor T cells by OCDC [97].

Important immune barriers in the OC TME, including VEGF, Treg cells and tumor endothelial Fas ligand (FasL) can drive tumor angiogenesis and hinder the functions of antitumor T cells [155,156]. Such immune barriers can potentially dampen cancer vaccine-induced T cell responses. Besides targeting Treg cells and VEGF, we also used acetylsalicylic acid to modulate tumor endothelial FasL expression and low-dose IL-2, which supports in vivo tumor-specific T cell proliferation. In the ovarian ID8 model, we demonstrated that mice treated with all these therapeutic agents in combination of OCDC had reduced tumor burden and survived significantly longer [97]. Reduced tumor burden was associated with an increase priming of specific tumor neoantigen-reactive T cells. Significant increases in perforin-expressing CD3^+^ and CD8^+^ TILs as well as reductions in tumor-infiltrating Treg cells were also observed in the tumors, indicating a more favorable TME for antitumor T cells to function. Adding acetylsalicylic acid led to reduced tumor endothelial FasL expression, which FasL was implicated in the preferentially killing of CD8^+^ TILs through Fas-FasL interaction [156]. We found a correlation between an increasing number of CD8^+^ TILs and reducing number of FasL^+^ tumor endothelial cells. Previously, we demonstrated the tumor endothelial FasL was induced by tumor-derived VEGF, IL-10, and prostaglandin 2 (PGE2) and the combined use of anti-VEGF antibody and acetylsalicylic acid helped to attenuate FasL expression through inhibiting VEGF and PGE2; these interventions substantially increased infiltration of CD8^+^ TILs [156]. These results indicated that already available immunomodulatory agents (IL-2 and acetylsalicylic acid) and OC standard-of-care can modulate the OC TME to facilitate the priming of antitumor T cells by personalized cancer vaccines and ensure a stronger overall efficacy.

ICB therapy can complement personalized cancer vaccinations by counteracting the inhibitory signals of T cell activation for tumor-specific T cells to mount a durable immune response [157]. Two anti-PD-1 antibodies, nivolumab and pembrolizumab, are approved by the FDA as frontline treatment in metastatic melanoma and have gained fast-track approval in many indications but not in OC. Nevertheless, nivolumab (a fully human immunoglobulin G4 [IgG4] anti-PD-1 monoclonal antibody) and pembrolizumab (a humanized anti-PD-1 IgG4 antibody) have been evaluated in OC [158,159]. OC patients who received nivolumab at 3 mg/kg showed a better overall response rate (RR) of 20% compared to patients who received 1 mg/kg (RR = 10%). Two patients receiving the higher dose showed complete response (CR) [158]. OC patients treated with pembrolizumab experienced stable diseases (6 out of 26), and two patients had partial responses while one patient had CR. A total of 23.1% of the patients showed evidence of tumor reduction [159]. The effectiveness of ICB therapy in OC is insufficient and could be enhanced with cancer vaccination, as both therapies sought to augment antitumor T cell responses. We previously demonstrated that the combinatorial use of a cancer vaccine, anti-PD-1 and anti-CTLA-4 led to improved overall survival in ID8 tumor-bearing mice [160]. Double blockade with anti-PD-1 and anti-CTLA-4 antibodies led to increased proliferation of antigen-specific T cells and inhibition of suppressive Treg cells. The further combination with a dose of GM-CSF-secreting irradiated ID8-VEGF tumor cell vaccine resulted in tumor rejection in 75% of the mice [160]. Several clinical trials are evaluating the combinatorial use of first-line chemotherapy and ICB therapy (NCT03734692, NCT03959761, NCT03598270, NCT03539328, NCT03170960, NCT04042116; www.clinicaltrials.gov (accessed on 2 September 2021)). These trials will yield important information on optimal timings for adding cancer vaccines in this setup.

ICB can also potentially synergize with PARP inhibitors, given that the latter causes cumulative chromosomal rearrangements that can increase mutation burden and tumor antigen presentation. It is demonstrated that a higher tumor mutational load is associated with an enhanced efficacy in ICB therapy in non-small cell lung cancer [161]. Furthermore, the presence of mismatch repair deficiency in colorectal cancer (Lynch-Syndrome that is similar to *BRAC* mutation) is strongly correlated with successful ICB therapy [162]. In a *Brac1*-knockout ID8 tumor model, the combined use of PARP inhibitor veliparib and anti-CTLA-4 resulted in prolonged survival of the mice compared to using veliparib alone. This was due to elicitation of memory T cell responses [163]. Hence, the combined use of PARP inhibition, ICB therapy and cancer vaccines in OC is warranted. Immunomodulatory agents such as acetylsalicylic acid and low-dose IL-2 that can modulate OC TME should also be considered in the combinatorial strategy.

## 4. Preclinical Ovarian Tumor Animal Models as Tools for Clinical Translation

The use of tumor animal models is essential for understanding and elucidating the complex molecular and genetic pathways in cancer pathology. Furthermore, tumor models are especially important for elucidating the dynamics of the tumor microenvironment and unraveling the complex interplay between cancer pathogenesis and immune system; an intact immune system is required as it could not be recapitulated in ex vivo cell culture systems. Different types of tumor models, such as synergic, patient-derived xenograft (PDX) and genetically engineered mice have been developed for OC research. As HGSOC is the most prevalent EOC subtype (~90%), we focus on tumor models that are developed for this subtype. In this section, we discuss the advantages and disadvantages of each tumor model as well as any animal studies that described the use of cancer vaccines in combination with OC standard-of-care.

### 4.1. Syngeneic ID8 Tumor Model

The murine ovarian ID8 tumor model is a well-characterized and commonly used syngeneic tumor model of OC. It shows a similar pathology to advance stage III and IV human HGSOC that is characterized by disseminated tumors in the peritoneal cavity and hemorrhagic ascites fluid formation [164]. The ID8 tumor cell line is developed by prolonged passage of the C57BL/6 murine ovarian surface epithelial cells (MOSEC) ex vivo and shows the ability to induce high tumor load following peritoneal implantation in C57BL/6 mice [164]. ID8 tumor cells can be implanted intraperitoneally, subcutaneously or orthotopically to generate a disseminated peritoneal carcinomatosis or a localized disease. The major advantage of an ID8 syngeneic mouse model is that both the implanted tumor cells and immunocompetent host are of the same genetic background; this enables us to study the effectiveness and interactions of different immuno-oncologic drugs with an intact immune system.

We have used the ID8 tumor model extensively for evaluating cancer vaccination strategies. Previously, we adapted our human OCDC generation protocol to produce the mouse equivalent OCDC for in vivo evaluation [143]. We demonstrated that both human and mouse OCDCs were capable of eliciting antigen-specific T cells and led to a reduction of sera IL-10 in the OC patients and mice bearing ID8 peritoneal carcinomatosis [143]. We also used the ID8 tumor model to evaluate the combinatorial use of OCDC, acetylsalicylic acid, low-dose IL-2 and OC standard-of-care [97]. Using a therapeutic schedule that closely mimicked the regimens in our phase I OC clinical trial, we demonstrated strong similarities between the treated mice and OC patients in terms of prolonged overall survival and elicitation of polyclonal tumor neoantigen-specific T cells. We also demonstrated efficacy with a combinatorial strategy consisting of a GM-CSF-secreting tumor cell vaccine and ICB therapy (anti-PD-1 and anti-CTLA-4) in mice bearing ID8-VEGF-expressing tumor. Hence, these results suggested that the ID8 tumor model is a relevant and useful model for investigating immunotherapeutic approaches for OC clinical translation. Morse et al. orthotopically implanted ID8-VEGF-expressing tumor cells beneath the ovarian bursa of C57BL/6 mice to model micro-metastatic OC disease that may be useful for evaluating combinatorial therapies in a minimal disease setting [165].

**Figure 2:**
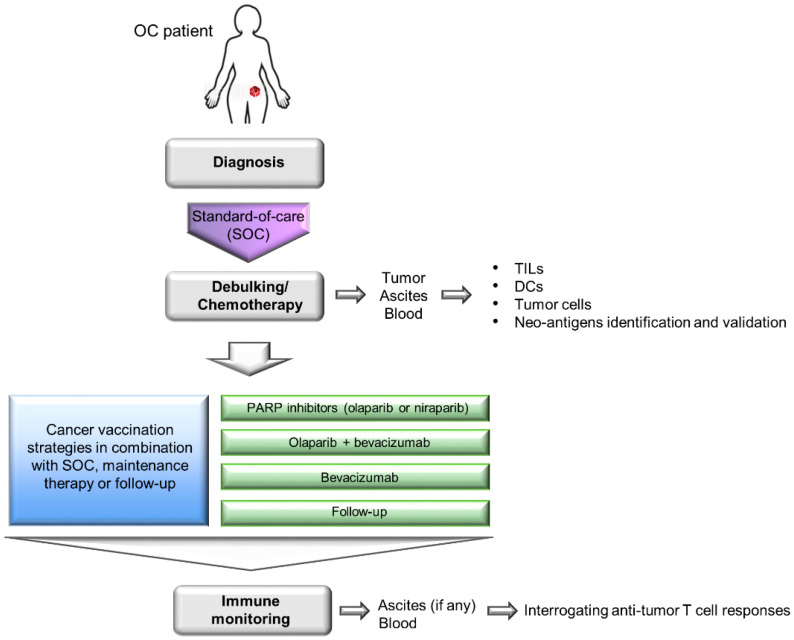
Proposed cancer vaccination strategies in the consolidation setting following standard-of-care chemotherapy and surgery in OC. The preclinical murine ID8 ovarian tumor model can serve as a useful tool to mimic the actual OC clinical scenario described here for evaluating combinatorial OC standard-of-care treatments and cancer vaccination strategies. As an example, during the first-line debulking surgery, different clinical samples (i.e., tumors, ascites and blood) can be obtained from the patients for preparing tumor WTL, DC vaccine, identifying and validating tumor (neo)-antigens from TILs and tumor cells. Next, different cancer vaccination strategies as described in Figure 1 can be implemented in combination with the OC standard-of-care, maintenance therapy or follow-up. Finally, additional blood and ascites samples can be obtained from the patients to evaluate their therapeutic anti-tumor T cell responses as well as track their disease regressions.

A major criticism of the wild-type ID8 tumor line is that it does not harbor any pathogenic mutations commonly seen in human HGSC (e.g., mutations in *Tp53* [166], *Brca1* or *Brca2* [21,22,23] genes). To model these gene defects, three derivative ID8 murine cell lines deleted of *Tp53*, *Brca1* and/or *Brcac2* genes were generated via CRISPR/Cas9 gene editing [167,168]. The loss of *Tp53* gene led to faster tumor growth and increased CCL2 expression that promoted immunosuppressive myeloid cells infiltration into primary tumors and ascites. Furthermore, ID8 deleted of both *Tp53* and *Brca1* genes was responsive to PARP inhibitor rucaparib, showed CD3^+^ TILs in the primary tumors and slower tumor growth [167]. These findings suggest that these derivative ID8 tumor lines could closely mimic the OC patient populations in clinics who require different first-line treatment strategies (Figure 1). These derivative ID8 tumor lines should help to facilitate a more accurate evaluation of the treatment outcome in the context of immunotherapy-standard-of-care combinations. In a preliminary study, we observed that mice bearing these tumors showed prolonged overall survival following OCDC vaccinations (unpublished data). The next step will be to incorporate OC standard-of-care and other immunomodulatory therapies in these ID8 tumor models, and closely follow the patient treatment regimens outlined in Figure 2.

### 4.2. Orthotopic Patient-Derived Xenograft (PDX)

PDX models are generated by implanting patient-derived materials, such as primary tumor tissues, ascites fluid or established OC tumor lines into immunocompromised mice (e.g., athymic nude mice or severe combined immunodeficiency (SCID) mice). The assumption is that a PDX model allows the preservation of tumor heterogeneity and molecular features that are associated with human OC and should facilitate the study of tissue site-specific pathology and metastasis. One caveat of using established OC tumor cell lines, such as SK-OV-3 and OVAR5, in a PDX model is that prolonged ex vivo passages rendered them genetically different from the original parent and primary OC tumors. Conversely, the use of primary tumor pieces from patients in PDX models will better reflect the genomic mutations potentially observed in the patients. Orthotopic implantation of primary tumor materials into clinically relevant organ sites can also yield higher predictive results. As immunocompromised mice are used in PDX models, evaluating cancer vaccination strategies is not feasible, as a functioning immune system is required. Nevertheless, PDX models are useful for drug testing (e.g., chemotherapy) to help identify optimal drug combinations in patients. We have successfully used PDX models to evaluate the in vivo cytolytic capacity of OCDC-primed T cells isolated from the peripheral blood of vaccinated OC patients [97]. Ex vivo generated TILs have also been evaluated for their reactivity against patient-matched autologous tumor cells in an ovarian PDX model and demonstrated the ability to produce IFN-γ in an HLA-dependent manner [169]. Hence, PDX models can help to generate useful information for OC trial designs.

Advances have been made in engineering humanized mouse models that can accept human fetal liver or adult CD34^+^ multipotent hematopoietic stem cells (HSCs) and support the development of a functional human innate immune system from injected HSCs. A mouse strain MI(S)TRG has been developed to harbor human genes encoding for macrophage-colony stimulating factor (M-CSF), IL-13, GM-CSF and thrombopoietin to support the development of human monocytes, macrophages and natural killer cells from progenitor cells [170]. Further analysis showed that the human macrophages are able to infiltrate the human tumor xenograft in a pattern similar to that observed in primary tumors [170]. Another humanized mouse model has been created by engrafting primary ovarian tumor tissues containing TILS and tumor-associated fibroblasts intraperitoneally into the non-obese diabetic (NOD)-scid IL2rγ^null^ (NSG) mouse [171]. Similar tumor progression is observed between these NSG mice and OC patients, as well as ascites formation and increasing levels of sera and ascites CA125 [171]. The NSG-SGM3 mouse expressed human hematopoietic stem cell factor GM-CSF and IL-3, which can effectively support the engraftment of human OC tumors and immune cells [172]. This mouse strain showed increased numbers of myeloid and Treg cells; these immune cells are known to be immunosuppressive in OC, and this model can be useful for modulating these cell populations with different immunotherapies. As the humanized PDX tumor models continue to improve, this platform can play an important role in evaluating personalized cancer therapy.

### 4.3. Genetically Engineered Mouse Models (GEMMs)

Numerous GEMMs have been developed for HGSC to gain a deeper understanding of its origin, pathogenesis and genetic mutations (reviewed in [173,174]). However, most of the GEMMs are generated on a mixed mouse strain background and unsuitable for evaluating tumor immunity and immunotherapeutic strategies. Moreover, the disease can develop over a wide timeframe, making it difficult to control tumor onset and outgrowth in the GEMMs. Nevertheless, recent efforts have been made to characterize and compare the TME of six different syngeneic mouse HGSC lines established from GEMMs to that of OC patient biopsies [175]. The authors selected tumor models that developed metastases in the omentum, a common site of OC metastasis, and identified features that were similar to human HGSOC, including cellular and molecular properties, innate and adaptive immune responses, and matrisome components [175]. These tumor models also exhibited common and distinct features in TMEs, making them potential tools for studying the responses of different subgroups of HSGOC patients to specific therapies.

## 5. Conclusions

Although advances have been made in OC standard-of-care, overall survival remains poor. Cancer vaccines have demonstrated effectiveness in OC patients and can be considered for potential incorporation into OC standard-of-care regimen. Using the murine ID8 ovarian tumor model, we demonstrated that the combinatorial use of a personalized cancer vaccine (OCDC), immuno-modulatory agents and OC standard-of-care led to greater overall efficacy. The syngeneic ID8 model is a well-characterized and useful model for human HGSOC for clinical translation; we are able to demonstrate several important similarities between this model and OC patients in terms of responses to immunotherapies. Other tumor models, including PDX and GEMMs, are continuing to improve and may serve as important tools for evaluating cancer vaccines and combinatorial therapies in the near future.

## Figures and Tables

**Table 1:** Completed OC clinical trials that investigated cancer vaccine strategies. Sources obtained from www.clinicaltrials.gov (accessed on 2 September 2021).

NCT ID	Cancer Type(s)	Treatments	No. of Patients Enrolled	Study Outcome	References
Tumor-Associated Antigen (TAA) Peptides					
NCT02498665	Ovarian cancer (OC), prostate cancer (PC), non-small cell lung carcinoma (NSCLC), renal cell carcinoma (RCC), sarcoma, melanoma, acute myeloid leukemia, myelodysplastic syndromes, glioblastoma multiform	WT1 protein-derived peptide vaccine (DSP-7888)	24	4 stable disease (SD), 16 progressive disease (PD) and 4 not evaluated. Overall survival (OS) was 180 months, and median progression-free survival (PFS) was 52 months	[51]
NCT02270372	OC, breast cancer (BC)	Peptide vaccine incorporating a synthetic glycolipopeptide MUC1 antigen (M40Tn6) and novel synthetic toll-like receptor (TLR)-4 agonist (PET lipid A) in a liposomal formulation (ONT-10), varlilumab	28	No published results	na
NCT00019084	OC, BC, CRC, PC, cervical cancer, lung cancer	Mutant p53/RAS peptide-pulsed DC vaccine, sargramostim, therapeutic autologous tumor-infiltrating lymphocytes	17 BC, 13 OC	OC patients showed median OS of 40.8 and 29.6 months for arm A and B, respectively, and the median PFS were 4.2 and. 8.7 months for arm A and B, respectively.	[52]
NCT00019916	OC, BC	p53 peptide, aldesleukin (recombinant IL-2)	21	13 and 7 patients received subcutaneous (SQ arm) and intravenous (IV arm) vaccination, respectively. The mean OS on the SQ arm was 70.4 months and on the IV arm was 72.9 months.	[53]
NCT01095848	OC, BC, PC	vaccine containing 7 tumor-specific HLA-A2-restricted peptides and a universal T-helper peptide, liposome and Montanide ISA51 VG (DPX-0907)	22	14 PD; patients were within the median progression-free survival period for their previous treatment	[54]
NCT01416038	OC, fallopian cancer, peritoneal cancer	DPX-Survivac (targeting survivin antigen), low dose cyclophosphamide	19	12 of 18 patients (67%) remained without clinical progression at 6-months follow up	[55]
NCT01580696	OC, fallopian cancer, peritoneal cancer	Folate-binding protein (FBP) epitope E39 peptide (100–500 mcg), sargramostim, an attenuated peptide of E39 (J65) as booster	51	Disease-free survival (DFS) improved in the 1000 μg group after treatment of primary disease (90.0% vs. CG: 42.9%, *p* = 0.007), but not in recurrent patients.	[56]
NCT02019524	OC, BC	E39 peptide, J65 vaccine	39	Increase in E39-specific cytolytic T cells (CTLs) were detected following vaccination and both epitopes were safe	[57]
NCT00003002	OC, BC, lung cancer	HER-2/neu peptide, sargramostim (recombinant GM-CSF)	60	No published results	na
NCT00005023	OC, BC, lung cancer	HER-2/neu peptide, sargramostim	15	Significant increases in patients’ pre- to post-vaccine delayed-type hypersensitivity (DTH) responses that were correlated with peptides vaccine doses. A reduction of GM-CSF did not affect DTH responses.	[58]
NCT00091273	OC, peritoneal cancer	Multipeptide (MAGE-A, FBP and HER-2/neu) vaccine, sargramostim, incomplete Freund’s adjuvant	9	Most frequent side-effects: injection site pain, fatigue and head ache	[59]
NCT00616941	OC, fallopian tube cancer, primary peritoneal cancer	NY-ESO-1 overlapping peptide (OLP)4 emulsified in Montanide ISA51, Poly-ICLC	28	Six patients showed no evidence of disease (NED), and PFS ranging from 17–46 months	[60]
NCT00066729	OC, fallopian tube cancer, primary peritoneal cancer	NY-ESO-1 peptide vaccine, incomplete Freund’s adjuvant	9	Median time of disease progression/recurrence from start of vaccination: 19.0 mo, 1 patient complete regression of metastatic disease after 10 immunizations	[61]
NCT02764333	OC	Proteins derived from the folate receptor-alpha admix with GM-CSF (TPIV200), durvalumab	27	Median OS of 21 months, and median PFS of 2.8 months	[62]
NCT00437502	OC, fallopian tube cancer, primary peritoneal cancer	Multipeptide vaccine, Montanide ISA-51, sargramostim	8	Median OS not reached	[63]
NCT00939809	OC, fallopian tube cancer, primary peritoneal cancer, ovarian clear cell cystadenocarcinoma, ovarian endometrioid adenocarcinoma	Urokinase-Derived Peptide A6	31	Median PFS was 2 months, and 1 hemorrhage death possibly related to study	[64]
NCT01606241	Recurrent OC, BC, fallopian tube cancer, primary peritoneal cancer	Multiepitope folate receptor-alpha peptide vaccine, cyclophosphamide	22	Median PFS was 528 days (~17.6 months) in patients who were in first remission. Median OS not reached for patients who were in second remission.	[65]
NCT01673217	Recurrent OC, fallopian tube cancer, primary peritoneal cancer	NY-ESO-1 peptide vaccine, pegylated liposomal doxorubicin hydrochloride, sargramostim, incomplete Freund’s adjuvant, decitabine	10	6 SD	[66]
NCT01485848	Recurrent OC	Synthetic targeted cytolytic peptide conjugated to luteinizing hormone-releasing hormone (LHRH)-alpha receptors on surfaces of tumor cells (EP-100)	44	No difference in response rate was detected with the addition of EP-100 to paclitaxel in the overall patient population	[67]
NCT01639885	Recurrent OC	p53-synthetic long peptide (SLP), IFN-α2b	15	4 SD, 2 partial response (PR), 10 PD following computerized tomography (CT) scan	[68]
NCT00844506	OC	p53-SLP, cyclophosphamide	10	2 SD	[69]
NCT00006041	OC, fallopian tube cancer, peritoneal cancer	MUC1-keyhole limpet hemocyanin (KLH) conjugate vaccine, adjuvant QS21	11	8 of 9 patients developed responses against to at least 3 different tumor antigens	[70]
NCT01248273	OC, fallopian tube cancer, primary peritoneal cancer	Globo-H-GM2-sTn-TF-Tn-KLH conjugate, adjuvant QS21	25	Median PFS of 6 months, and 5 patients remained in complete clinical remission (CCR) at 18-months follow up	[71]
NCT03332576	OC, fallopian tube cancer, primary peritoneal cancer	DPX-Survivac, low dose cyclophosphamide	19	No published results	na
NCT00857545	Stage I-IV OC, fallopian tube cancer, primary peritoneal cancer	Polyvalent vaccine-KLH conjugate vaccine (i.e., Globo-H-KLH, Tn-mucin 1 [MUC1]-32mer-KLH, and Thompson Friedreich antigen [TF]-KLH plus OPT-821), saponin-based immunoadjuvant OBI-821	171	KLH + OPT-821 was not superior to OPT-821 alone (hazard ratio [HR]: 0.98; 2-sided 95% CI, 0.71–1.36). The median OS for KLH + OPT-821 and OPT-821 were 47 and 46 months, respectively.	[72]
NCT01003808	Solid tumors	Peptide vaccine containing nanoparticles of cholesteryl hydrophobized pullulan [CHP] complexed with the cancer-testis antigen NY-ESO-1 protein (IMF-001)	25	No tumor shrinkage was observed. Patients receiving 200 μg of CHP-NY-ESO-1 survived longer than patients receiving 100 μg of CHP-NY-ESO-1, even those who exhibited unresponsiveness to previous therapies or had higher tumor burdens.	[73]
NCT01617629	OC	Peptide cancer vaccine containing nanoparticles of cholesteryl hydrophobized pullulan [CHP] complexed with the cancer-testis antigen NY-ESO-1 protein (CHP-NY-ESO-1 peptide vaccine IMF-001)	28	4 patients showed CA125 response or stabilization	[74]
NCT00005956	OC, BC, gastric cancer (GC)	HER-2/neu intracellular domain protein-pulsed DC vaccine	9	1 SD for 3 months, and showed 1 tumor size reduction	[75]
RNA and DNA vaccines (alone or DC-based)					
NCT00004604	OC, BC, CRC, GC, hepatocellular carcinoma (HCC), PC, gallbladder cancer, extrahepatic bile duct cancer, head and neck cancer, testicular germ cell tumor	Carcinoembryonic antigen (CEA) RNA-pulsed DC cancer vaccine	24	1 complete response (CR), 2 PR, 3 SD and 18 PD	[76]
NCT01322802	Stage III-IV OC and ovarian germ cell tumor	A multiepitope plasmid DNA vaccine containing mammalian expression vector pUMVC3, encoding epitopes of human insulin-like growth factor-binding protein 2 (hIGFBP-2) [pUMVC3-Higfbp-2 vaccine]	25	OS rate at 2-years was 82%	[77]
NCT01118052	Recurrent OC, fallopian tube cancer, primary peritoneal cancer	PEG-PEI-cholesterol lipopolymer-encased IL-12 DNA plasmid vector (GEN-1)	16	7 SD, 9 PD, median PFS and OS were 2.89 and 9.17 months, respectively	[78]
NCT00381173	OC, BC, colorectal carcinoma (CRC), PC and RCC	Plasmid DNA encoding for cytochrome P450 Family 1 Subfamily B Member 1 (CYP1B1) and encapsulated in biodegradable poly-DL-lactide-coglycolide microparticles (ZYC300), cyclophosphamide	22	3 SD, PD observed in 10 patients who did not respond to CYP1B1	[79]
Viral vector vaccine					
NCT00408590	OC, primary peritoneal cancer	CEA-expressing oncolytic measles virus, oncolytic measles virus encoding thyroidal sodium iodide symporter (MV-NIS)	37	MV-NIS showed a median of OS 26.5 months, while MV-CEA showed a median OS of 12.15 months	[80,81]
NCT02275039	Recurrent OC, fallopian tube cancer, primary peritoneal cancer	Modified vaccinia virus ankara vaccine expressing p53, gemcitabine hydrochloride	11	3 SD, 1 PR, and PFS 3 months	[82]
NCT00602277	Recurrent OC, fallopian tube cancer, primary peritoneal cancer	Non-pathogenic isolate of the unmodified Reovirus (REOLYSIN^®®^; Pelareorep)	70	No published results (conference paper)	[83]
NCT00964756	OC	An infectivity-enhanced adenovirus expressing a therapeutic thymidine kinase suicide gene and a somatostatin receptor (Ad5.SSTR/TK.RGD), ganciclovir	12	5 SD, 7 PD	[84]
NCT02028117	Recurrent platinum-resistant OC	A group B Ad11p/Ad3 chimeric oncolytic adenovirus (Enadenotucirev)	No information	No published results (conference paper)	[85]
NCT01199263	Recurrent OC, fallopian tube cancer, primary peritoneal cancer	Pelareorep, paclitaxel	108	Median PFS of 4.4 months	[86]
NCT00562003	OC, primary peritoneal cancer	Replication-competent oncolytic adenovirus 5 carring a 24-bp deletion in E1A gene (Ad5-delta24RGD)	21	4 SD, 6 PD	[87]
NCT01536054	Recurrent OC, fallopian tube cancer, primary peritoneal cancer	Replication-defective recombinant canarypox virus [ALVAC(2)] encoding NY-ESO and the TRIad of COstimulatory Molecules (B7-1, intracellular adhesion molecule-1 [ICAM-1] and leukocyte function-associated antigen-3 [LFA-3]; also called TRICOM) (ALVAC(2)-NY-ESO-1 (M)/TRICOM vaccine), sirolimus, sargramostim	7	No published results	na
NCT02179515	OC, BC, PC, lung Cancer, other tumors	Replication-deficient, attenuated derivative of the vaccinia virus strain Ankara expressing a CD8+ T cell epitope of brachyury and TRICOM (MVA Brachyury-TRICOM)	38	34 of 38 patients completed all three doses of therapy; 21 PD and 17 SD observed.	[88]
NCT00004032	Recurrent OC	Canarypox viral vector carrying the gene for human B7.1 (CD80 antigen) (ALVAC-hB7.1), recombinant interferon gamma	No information	No published results	na
NCT00027534	OC, BC, CRC, GC, HCC, PC, gallbladder cancer, head and neck cancer, testicular germ cell tumor	Recombinant fowlpox virus vector encoding CEA and TRICOM (fowlpox-CEA-B7-1/ICAM-1/LFA-3rF-CEA(6D)TRICOM)	14	1 patient had a decrease in the CEA level, and 5 showed SD. CEA-specific T cells were detected in 10 patients.	[89]
NCT00028496	OC, BC, CRC, GC, HCC, PC, cholangiocarcinoma, ovarian endometroid adenocarcinoma	Fowlpox-CEA-B7-1/ICAM-1/LFA-3rF-CEA(6D)TRICOM, sargramostim	58	9 SD for 4 months, 14 SD for >6 months, 1 complete response (CR)	[90]
NCT00088413	OC, BC, CRC, adenocarcinoma	Recombinant vaccinia (PANVAC-V) and recombinant fowlpox (PANVAC-F) expressing MUC1, CEA and TRICOM, sargramostim	26	Median OS of 15.0 months	[91]
NCT00112957	OC, fallopian tube cancer, primary peritoneal cancer	Recombinant vaccinia-expressing NY-ESO-1 (rV-NY-ESO-1) and recombinant fowlpox-expressing NY-ESO-1 (rF-NY-ESO-1) vaccines	22	Median progression-free survival (PFS) was 21 months, and median OS was 48 months	[92]
NCT00803569	OC, fallopian tube cancer, primary peritoneal cancer	ALVAC(2)-NY-ESO-1(M)/TRICOM vaccine, sargramostim	No information	No published results	na
NCT03127098	OC, BC, PC, CRC, thyroid cancer	Virus expressing CEA (ETBX-011), IL-15 superagonist complex (ALT-803; ALT-803)	No information	No published results	na
Whole tumor lysate (WTL) or cell vaccine (alone or DC-based)					
NCT01312389	OC, fallopian tube cancer, primary peritoneal cancer	Autologous oxidized WTL (OC-L) emulsified with Montanide ISA 51 VG, Ampligen	No information	No published results	na
NCT01551745	Stage III-IV OC	Vigil™, bevacizumab	No information	No published results	na
NCT01867086	Stage III-IV OC	Vigil™ vaccine, carboplatinum, taxol	42	In the Vigil^®®^ arm, a PFS mean of 826 days (27.5 months) and median of 604 days (20.1 months) were observed. In the control arm, a PFS mean of 481 days (16 months) and median of 377 days (12.6 months) were observed.	[93]
NCT00478452	OC, fallopian tube cancer, primary peritoneal cancer	Autologous DCs pulsed with killed autologous tumor cells (DC-Ova), cyclophosphamide	11	6 NED at 36 months. The 3-years PFS was 80% and 3-years OS was 100%.	[94]
NCT00683241	OC, primary peritoneal cancer	Autologous tumor lysate-pulsed DCs (DCVac-L)	No information	No published results	na
NCT01068509	OC	Autologous DCs pulsed with mannosylated-MUC1 fusion protein (M-FP) (Cvac)	56	PFS of 13 months observed	[95]
NCT01132014	OC	Autologous DCs pulsed with oxidized WTL (OCDC)	67	2 PR, 14 SD	[96,97]
NCT03657966	Recurrent OC	Autologous DCs pulsed with allogeneic apoptotic tumor cells (DCVAC/OvC), OC standard-of-care chemotherapy	No information	No published results (conference paper)	[98]

Note: na denotes not available. Grey: to highlight and separate the different forms of cancer vaccines listed in the table.

**Table 2:** Current OC clinical trials that are investigating cancer vaccine strategies, including two registered trials with unknown study status. Sources obtained from www.clinicaltrials.gov (accessed on 2 September 2021).

NCT ID	Cancer Type(s)	Treatments	Status	No. of Patients to Enrol
Neoantigen peptides				
NCT04024878	Ovarian cancer (OC)	Neoantigen peptide vaccine, nivolumab	Recruiting	30
NCT04713514	Platinum-sensitive and recurrent OC	A multi-neoepitope vaccine covering relevant OC TAAs including p53 (OSE2101), pembrolizumab	Not yet recruiting	180
Tumor-associated antigen (TAA) peptides				
NCT02737787	Recurrent OC, fallopian tube cancer, primary peritoneal cancer	Wilms tumor 1 (WT1) and NY-ESO-1 peptides, nivolumab	Recruiting	20
NCT01376505	OC, breast cancer (BC), colorectal cancer (CRC), gastrointestinal stroma cancer (GIST)	HER-2/neu peptide	Recruiting	100
NCT03761914	OC, acute myelogenous leukemia, CRC, triple-negative BC, non-small cell lung cancer (NSCLC)	A multivalent multipeptides of >20 epitopes of WT1 protein (Galinpepimut-S), pembrolizumab	Recruiting	90
NCT04853017	Minimal residual disease, OC, CRC, NSCLC, pancreatic adenocarcinoma (PC), cholangiocarcinoma, bile duct cancer, gallbladder carcinoma	Lipid-conjugated oligonucleotide [Amph-CpG-7909] admixed with lipid-conjugated KRAS/NRAS-derived peptides [Amph-Peptides]) (ELI-002)	Recruiting	159
NCT00194714	HER-2/neu-positive stage IV OC or BC	HER-2/neu peptide	Active, not recruiting	26
NCT02111941	OC, fallopian tube cancer, primary peritoneal cancer, ovarian clear cell cystadenocarcinoma, ovarian endometrioid adenocarcinoma	Multiepitope folate receptor-alpha peptides-loaded DC vaccine	Active, not recruiting	19
RNA and DNA vaccines				
NCT04163094	OC	Liposome-formulated mRNA vaccine encoding for three OC TAAs (W_ova1), neoadjuvant chemotherapy	Recruiting	10
NCT00436254	HER-2/neu-positive stage III-IV OC, OC germ cell tumor, BC	DNA plasmid vaccine (pNGVL3-hICD), sargramostim (recombinant granulocyte-marcophage colony stimulating factor [GM-CSF])	Active, not recruiting	66
Viral vector vaccine				
NCT04246671	OC, BC, CRC, NSCLC, PC, hepatocellular cancer (HCC), gastric cancer (GC), chordoma, prostate cancer, Merkel cell carcinoma	Modified Vaccinia Ankara-BN (MVA-BN) viral vector vaccine expressing Brachyury and HER-2/neu proteins (TAEK-VAC-HerBy)	Recruiting	45
NCT03113487	Recurrent platinum-resistant OC, fallopian tube cancer, primary peritoneal cancer	Modified Vaccinia Virus Ankara viral vector vaccine expressing p53 protein, pembrolizumab, gemcitabine hydrochloride	Recruiting	28
NCT03120624	Recurrent OC, fallopian tube cancer, primary peritoneal cancer, endometrioid adenocarcinoma	Recombinant Vesicular Stomatitis Virus-expressing human interferon-beta and sodium-iodide symporter (VSV-hIFNbeta-NIS), ruxolitinib phosphate	Recruiting	77
NCT04282044	OC, triple-negative BC, CRC, HCC, GC, osteosarcoma	Activated cytokine-induced killer (CIK) cells infected with an oncolytic virus (CRX-100)	Recruiting	24
NCT03225989	OC, CRC, PC, biliary carcinoma	Oncolytic adenovirus expresses transgenes trimerized membrane-bound isoleucine zipper (TMZ) TMZ-CD40L and 41BBL (delolimogene mupadenorepvec; LOAd703)	Recruiting	50
NCT02364713	OC, fallopian tube cancer, primary peritoneal cancer	Mesenchymal Stem Cells infected with oncolytic Measles Virus encoding for thyroidal sodium iodide symporter (MV-NIS)	Recruiting	66
NCT02068794	OC, fallopian tube cancer, primary peritoneal cancer, endometrioid adenocarcinoma	Mesenchymal Stem Cells infected with MV-NIS	Recruiting	57
NCT03663712	Platinum-resistant ovarian cancer, stage IV peritoneal carcinomatosis	Type I genetically modified oncolytc Herpes Simplex Virus (Talimogene Laherparepvec; TVEC)	Recruiting	24
NCT02759588	OC, fallopian tube cancer, primary peritoneal cancer	Triple-modified and attenuated Vaccinia virus (Lister strain) [GL-ONC1], chemotherapy, bevacizumab	Active, not recruiting	64
Whole tumor lysate (WTL) or cell vaccine (alone or DC-based)				
NCT00722228	OC and solid tumors of stage II, III and IV	Autologous or allogeneic tumor cell vaccine	Recruiting	50
NCT03556566	OC	Tableted vaccine (V3-OVA) prepared from autologous hydrolyzed, inactivated blood and tumors.	Recruiting	20
NCT03671720	Advanced and metastatic cancers	Autologous dendritic cell (DC) pulsed with autologous whole tumor lysate	Recruiting	10
NCT04212377	Endometrial cancer	Myeloid and plasmacytoid DC (nDC) pulsed with WTL, MUC1 and survivin peptides	Recruiting	8
NCT04834544	OC, fallopian tube cancer, primary peritoneal cancer	Autologous DCs pulsed with allogeneic apoptotic tumor cells (DCVAC/OvC)	Recruiting	75
NCT03735589	Stage II fallopian tube cancer	Alpha-type-1 polarized dendritic cells pulsed with autologous tumor + autologous natural killer cell-like cytolytic T cells	Not yet recruiting	18
NCT03905902	OC, fallopian tube cancer, primary peritoneal cancer	DCVAC/OvCa, standard-of-care platinum-based chemotherapy (carboplatin, gemcitabine, paclitaxel, pegylated liposomal doxorubicin), bevacizumab	Not yet recruiting	678
NCT04614051	OC	Autologous DCs pulsed with ovarian cancer-specific antigen(s) (Cellgram-DC)	Not yet recruiting	10
NCT04739527	OC	Irradiated mature allogenic DCs (DCP-001)	Not yet recruiting	17
NCT01309230	OC	Modified autologous tumor cells expressing GM-CSF (Vigil™)	Active, not recruiting	44
NCT02033616	Stage III-IV OC, fallopian tube cancer, primary peritoneal cancer	Autologous DCs pulsed with autologous tumor cells	Active, not recruiting	99
NCT00660101	OC	2,4-dinitrophenyl (DNP) keyhole limpet hemocyanin-Modified autologous tumor cell vaccine (OVax^®®^)	Unknown status	34
NCT00703105	OC and solid tumors of stage II-IV	DCs pulsed with autologous WTL or HLA-A2-restricted MUC1 and WT1 peptides	Unknown status	36
DC-tumor cell fusion vaccine				
NCT00799110	OC, fallopian tube cancer, primary peritoneal cancer	DC-tumor cell fusion vaccine, sargramostim, imiquimod	Active, not recruiting	23

Grey: to highlight and separate the different forms of cancer vaccines listed in the table.
